# The Week's Work

**Published:** 1911-09-09

**Authors:** 


### The Week's Work.

### A WORKER FOR THE STREATHAM INCURABLES.

Since publishing our note of August 26 upon the hospital work of the late Mr. John Hampton Hale, we have received some interesting particulars concerning his connection with the British Home for Incurables at Streatham. He was best known as its treasurer, to which post he was appointed in 1884, but it was long previous to that—in 1870, in fact—that he first joined the Board. Time and experience eventually made him its senior member and deputy-chairman, and his enthusiasm for the Home continued unabated till his death. For instance, this year, when the jubilee of the British Home for Incurables was to take place, he spared no trouble with the arrangements for making that function a widely known public success.

### NEW HOSPITAL AT LITTLEHAMPTON.

The new buildings of the Littlehampton Hospital were opened by Lady Aubrey-Fletcher last week, on the site in Fitzallan Road which we understand has been given by the Duke of Norfolk. Mr. W. Beldam, chairman of the committee, explained to a large audience of visitors that seven years ago the town had no public means of mending broken ribs, except those of umbrellas, and so it was decided to take the £150 which had been subscribed to commemorate the Diamond Jubilee (no memorial for which had been found), and to use it as a nucleus of a Hospital Fund. This led, in spite of the grumblers, to the temporary conversion of the former house in Surrey Street. When enlargement became necessary it was decided not to extend but to rebuild. The Duke of Norfolk, who was an old subscriber, gave the site, and £2,008 has been spent upon the building. Four hundred more is needed to complete the furnishing. Mr. E. H. Cox, the honorary secretary, received the deed of gift, and together with the chairman and the committee of management he is to be congratulated on the successful stage of growth. The architects are Messrs. Wheeler and Godman, of Horsham.

### MUSIC AND A YORKSHIRE HOSPITAL

We publish elsewhere a very interesting letter on the subject of music as a source of income for the Yorkshire hospitals, which was sent to us as a result of a recent note in "Week's Work" on "Hospital Concerts in Germany." Though probably the strict form of village wake as an annual event is peculiar to Yorkshire, somewhat similar local customs abound throughout the shires, as all readers of Thomas Hardy's "Under the Greenwood Tree" will remember from his account of the peripatetic choirs of Wessex. Why should not Midland and Southern provincial hospitals follow the example of the Beckett Hospital in this matter? The letter referred to shows clearly how this may be done. It would not be the first time for the southern hospitals to benefit by the enterprising example of institutions in the North, as the adoption of Workers' Hospitals Funds (a North Country idea), for instance, in connection with Addenbrooke's Hospital, Cambridge, conclusively shows. We think our readers will appreciate Mr. Whitham's letter as a suggestion well worthy of their consideration. It is an example of the value of the interchange of hospital experience, which is what institutional solidarity really means.

### HOSPITALS AND PAYMENT OF MEMBERS.

It is curious that the first quarterly instalment of the much discussed payment of members should have set an example which may assist the voluntary hospitals in all the constituencies represented by rich men throughout the country. Mr. Arthur Fell, Conservative M.P. for Great Yarmouth, wrote a letter to last Saturday's papers from which we take the following: "I have to-day received a draft for £94 3s. 4d., which is described as the payment for my services as member of Parliament for the quarter ending June 30, 1911, less income-tax at 1s. 2d. in the pound. I have never received a payment which has been so repugnant to me as this . . . so I shall divide the money among the hospitals in the borough." We congratulate Mr. R. F. Ferrier, the honorary secretary, and Mr. Charles Orde, the treasurer, of the Great Yarmouth General Hospital on the useful quarterly addition to their hospital's income which they may now expect to receive. We hope from this that the voluntary hospitals throughout the country may assert their first claim to any such payment in all cases wherein the M.P. is known to be in no need of pecuniary assistance. This is the most unexpected and least controversial form of State aid for hospitals of which we have heard at present.

### EAST SUSSEX HOSPITAL COMPETITION.

As reported in The Hospital of August 26 (page 553), Mr. E. T. Hall, F.R.I.B.A., is appointed assessor in the above competition, which is open to all architects. £35,000 is the total sum available for the completed scheme, but it is not yet settled whether the scheme will be carried out in its entirety under one contract. The premiums offered are £125, £75, and £50. The suggestions by the Hospital Committee in the instructions include teak floors for wards, terrazzo for corridors, &c., all to have concave skirting; sash windows with hopper over. Electric wiring is to be hidden in all wards and public rooms in seamless steel paper-lined tubes to carry 50 volts or less; elsewhere the tubing may be exposed. A transformer will be required. To minimise fire risks two-inch hydrants are suggested at each end of large buildings, and these are to be connected with the main as well as to the storage tanks. Iron escape stairs external to all blocks, unless they have alternative means of escape internally. Plain unmoulded doors in oak or teak to parts used by patients. Balconies to the wards sufficiently large to accommodate beds. Sanitary towers built out from wards with ventilated gangways, to contain one bath, two w.c.'s, bed-pan sinks, two hand-basins, mackintosh sink and shelving, &c. Heating by radiators on low-pressure system. It is suggested that the wards would have a better appearance without centre block for fireplaces, but there may be indicated fireplaces at the ends or sides of the wards. Designs are to be delivered before the end of November, and the latest date for receiving questions is September 15.

### THE COMING CONFERENCE AT MANCHESTER.

By this time the majority of hospital officers will have received, as members, the invitation cards issued by the British Hospitals Association to the second annual Conference, which will be held at Manchester, on Thursday and Friday, September 28 and 29. On Thursday the meetings will be given up to a discussion of the Insurance Bill, in which Mr. Charles Lupton, Chairman of the General Infirmary, Leeds, Mr. Howard Collins, House Governor of the General Hospital, Birmingham, Dr. Mackintosh, M.V.O., Superintendent of the Western Infirmary, Glasgow, and Sir William Cobbett, Chairman of the Manchester Royal Infirmary, are expected to take part. On Thursday evening Mr. Charles Behrens, Lord Mayor of Manchester, has kindly invited the members to a reception in the Town Hall, where, by the way, the meetings will be held. On Friday, September 29, Dr. E. S. Reynolds, F.R.C.P., Chairman of the Medical Board, Manchester Royal Infirmary, and Mr. Charles Hopkinson, a member of the board, will read papers on the Representation of Medical Staffs on Boards of Management, and on the Methods of Distribution of Saturday and Sunday Funds respectively. On Saturday, we understand, through the courtesy of the directors of the Manchester Ship Canal, members will have an opportunity of viewing the canal from a steamboat. The railway companies have also provided special rates for members travelling to the Conference. There is little doubt, in short, that the success of last year's Conference at Glasgow will be repeated. Mr. W. G. Carnt, Superintendent of the Manchester Royal Infirmary, is acting very kindly as local honorary secretary, and he and Mr. C. W. Thies, hon. treasurer, are much to be thanked, as is the Lord Mayor also, for the trouble which they have taken to ensure the pleasure and profit of every member.

### SOME QUEER DEFINITIONS IN HOSPITAL DIET.

Recently interviewed by the *Sunday School Chronicle*, Mr. Conrad Thies, the retiring Secretary of the Royal Free Hospital, is reported to have given some curious illustrations of the sense in which the word food and the word luxury are interpreted by certain London hospitals. When he came to this hospital beer was looked upon as a food, and was largely used in all hospitals as a necessary article of diet.

"I can give you some interesting figures on this point. In 1887, the year in which I joined the hospital staff, out of a total expenditure of less than £11,000, the wine and spirit bill came to £267; in addition to this, £125 was spent on porter, ale, and stout, while the milk supplied cost only £354. Compare these figures with those for 1910, when there was a larger number of patients, and when the total expenditure exceeded £17,000. On milk we spent £938, on wines and spirits £57, and on malt liquor £9. The contrast speaks for itself, and we are not peculiar in this respect. At all the hospitals the milk bill has grown and the alcohol bill has gone down. It is rather an interesting fact in this connection," continued Mr. Thies, "that while beer and spirits in former days were regarded as food in the hospitals, tea, sugar, and butter were regarded as luxuries, and were not provided for the patients. They could have alcohol *ad lib.*, but if they wanted tea, sugar, or butter they had to buy it themselves. Of course, if they were too poor to afford these 'luxuries,' they were purchased for them out of the Samaritan Fund, but they did not appear on the diet sheet. Those were the circumstances when I came here, and I at once drew attention to the anomaly, and eventually the Board agreed to add tea, sugar, and butter to the diet sheet. This cost us about £200 a year, but the alcohol bill correspondingly decreased.

"It is curious, however, that at one or two of the largest hospitals in London this old arrangement is still in force. The medical officers may order champagne, expensive meat extracts, or alcoholic liquors, and they are readily provided; but no patient may have tea, sugar, or butter, except at his own expense. Why this practice is still adopted I cannot tell. To me it seemes absolutely indefensible."

We believe this verbal distinction formerly prevailed at the London Hospital, and whether or not the practice that results from it still survives, it would be interesting to hear Mr. Sydney Holland's account of the origin of the practice and the reasons which led to its continuance. In any case Mr. Thies's figures are well worth preserving, for they typify a curious and interesting phase in the development of the modern hospital.

### THIS WEEK'S DRUG MARKET.

Business in the drug market has improved to some extent since last report appeared, but there is still room for further improvement. Most of the changes in value are in an upward direction. Ipecacuanha is dearer. The price of ergot of rye is well maintained, and the drug is scarce. Citric acid continues to be in large demand and prices have advanced slightly. A good business has been done in cascara sagrada at increasing prices. Importers of quicksilver have reduced the price, and it is probable that makers of calomel, corrosive sublimate and other salts of mercury may make a corresponding reduction in the prices of these articles. Oil of aniseed is dearer, as also is oil of cloves. Higher prices are quoted for oil of peppermint. Cod-liver oil is dearer for the time being. The value of opium is well maintained, and there is no change in the position of morphine or codeine. The price of senega shows an advancing tendency. Sugar of milk is dearer. The price of carbolic acid is well maintained. Zinc oxide is dearer. The price of strychnine has advanced. Extremely high figures are quoted for otto of roses owing to the poorness of the crop.

